# Evidence-Based Public Health

**DOI:** 10.1155/2016/5681409

**Published:** 2016-01-28

**Authors:** Stefan K. Lhachimi, Malgorzata M. Bala, Giedrius Vanagas

**Affiliations:** ^1^Cooperative Research Group for Evidence-Based Public Health (ebph), Institute for Public Health and Nursing Research, University of Bremen and Leibniz Institute for Prevention Research and Epidemiology-BIPS, Achterstraße 30, 28359 Bremen, Germany; ^2^Health Sciences Bremen, University of Bremen, Bibliothekstraße 1, 28359 Bremen, Germany; ^3^Institute of Health Service Research and Health Economics, Faculty of Medicine, Heinrich-Heine University Düsseldorf, 40225 Düsseldorf, Germany; ^4^Department of Hygiene and Dietetics, Jagiellonian University Medical College, 31-034 Krakow, Poland; ^5^Department of Preventive Medicine, Lithuanian University of Health Sciences, LT-49264 Kaunas, Lithuania

Public health decision making can be a complicated process because of the complex nature of inputs and the need for group decision making. Nevertheless, public health research and practice during the last century gained many notable achievements and contributed to the 30-year gain in life expectancy. Despite these accomplishments, a greater attention to evidence-based approaches may be helpful. In its most straightforward definition, evidence-based public health (EBPH) means applying the principles of evidence-based medicine (EBM) to the field of public health [1]. However, the randomized clinical trials—the gold standard in EBM—are not always applicable in investigating public health problems. Key components of a broader definition of EBPH include making decisions on the basis of the best available scientific evidence by using sound data collection and research methods while engaging with the affected community in decision making. An evidence-based approach to public health could potentially have numerous direct and indirect benefits, including access to more and higher-quality information on best practice, a higher likelihood of successful prevention programs and policies, greater workforce productivity, and more efficient use of public and private resources.

In this special issue we collect several contributions to the emerging field of evidence-based public health, covering different aspects of the EBPH policy cycle, that is, from evidence generation and evidence synthesis to evidence communication and policy recommendation [2]. We are particularly delighted about the broad geographical coverage and methodological range of the included paper. The utilized methodology ranges from randomized controlled trial (RCT) to simulation studies with observational studies still being the most popular research design. The study titled “Worksite Tobacco Prevention: A Randomized, Controlled Trial of Adoption, Dissemination Strategies, and Aggregated Health-Related Outcomes across Companies” by V. Friedrich et al. conducted an RCT to compare various approaches to worksite tobacco prevention. An instructive finding of this study is that special attention must be paid to the dissemination of findings if adoption of proven public health measure should increase. Similarly, the article “Strong Public Health Recommendations from Weak Evidence? Lessons Learned in Developing Guidance on the Public Health Management of Meningococcal Disease” by G. Hanquet et al. shows the necessity to be transparent about the quality of the underlying evidence when giving policy recommendations.

Systematic reviews and meta-analysis are a workhorse of evidence-based medicine and two studies in our special issue demonstrate clearly that this is also the case for evidence-based public health. G. A. Kelley et al. conducted a systematic review titled “Exercise and BMI in Overweight and Obese Children and Adolescents: A Systematic Review and Trial Sequential Meta-Analysis” utilizing a trial sequential meta-analysis approach. This approach combines conventional meta-analysis methodology with meta-analytic sample size considerations; inferences derived from this method may potentially improve reliability of estimates. The systematic review “Sexual Risk Behaviors and HIV Infection among Men Who Have Sex with Men and Women in China: Evidence from a Systematic Review and Meta-Analysis” by H.-Y. Wang et al. sheds light on potential transmission mechanism of HIV/AIDS in China, indicating that more emphasis must be put on prevention measures for men who have sex with men and women.

A crucial, but at times overlooked, factor for a successful public health policy is the availability of trained medical staff. In our special issue two articles study this important topic. Interestingly enough, both are from Sub-Saharan Africa, a region that constantly faces the challenge of brain drain, that is, the outmigration of well-trained individuals. The study from Ethiopia titled “The Prevalence of Skilled Birth Attendant Utilization and Its Correlates in North West Ethiopia” by M. Alemayehu and W. Mekonnen looks at the prevalence of skilled birth attendants demonstrating that both social and technical factors must be addressed if this prevalence should be improved in the future. The study titled “Working Atmosphere and Job Satisfaction of Health Care Staff in Kenya: An Exploratory Study” by K. Goetz et al. investigates the factors that influence job satisfaction for health care works in Kenya. Clearly, improving job satisfaction may prove to be a very cost-effective tool in increasing retention rates among health care staff in Africa.

The contribution “Intervention Mapping to Adapt Evidence-Based Interventions for Use in Practice: Increasing Mammography among African American Women” by L. Highfield et al. uses the topic of mammography among African American women to demonstrate the use of intervention mapping as a tool for a systematic planning process. Their use of a simplified framework (IM Adapt) allowed adapting and implementing an evidence-based intervention to help underserved African American women to keep appointments for mammography screening. A separate paper titled “Evaluation of the Effectiveness and Implementation of an Adapted Evidence-Based Mammography Intervention for African American Women” by L. Highfield et al. evaluated the effectiveness of the identified intervention and added evidence to the finding that sequentially measuring efficacy and effectiveness of an evidence-based intervention, followed by implementation, may be missing important contextual information.

A paper from Croatia titled “Perinatal Health Statistics as the Basis for Perinatal Quality Assessment in Croatia” by U. Rodin et al. describes how the national perinatal health audit improved after introducing reporting criteria as recommended by WHO and PERISTAT. This database now allows comparison in perinatal outcome with other countries and targeting areas of improvement in a more evidence-based fashion. The contribution “Health Impacts of Increased Physical Activity from Changes in Transportation Infrastructure: Quantitative Estimates for Three Communities” by T. J. Mansfield and J. M. Gibson utilizes the approach of quantitative health impact assessment to assess the implications of increased physical activity from changes in transportation infrastructure. To this end they use a simulation tool (DYNAMO-HIA) that was specifically developed for such applications.

An Australian paper titled “Public Concern about the Sale of High-Caffeine Drinks to Children 12 Years or Younger: An Australian Regulatory Perspective” by C. M. Pollard et al. presents a study connected to the epidemiology of caffeine intake. Usually this is attributed mostly to coffee consumption. However, this paper demonstrates that growing concerns regarding the consumption of caffeinated beverages such as energy drinks by children and adolescents exist in communities in Western Australia, in particular by females and those living with children. These concerns increase with age. The final paper of this special issue titled “Smoke-Free Workplaces Are Associated with Protection from Second-Hand Smoke at Homes in Nigeria: Evidence for Population-Level Decisions” by D. Kaleta et al. aims to support decision making at the population level. These authors find evidence that smoke-free workplaces have the important additional effect of stimulating smoke-free homes in Nigeria and in turn reduce second hand smoking exposure of children.

In summary such collection of papers covering different issues relevant to EBPH will provide evidence for decision makers and will contribute to the development of this field.
